# Quasireversible Process of Dopamine on Copper-Nickel Hydroxide Composite/Nitrogen Doped Graphene/Nafion Modified GCE and Its Electrochemical Application

**DOI:** 10.1155/2014/724538

**Published:** 2014-06-16

**Authors:** Chuan-yin Liu, Zhong-yong Liu, Rong Peng, Zhi-cheng Zhong

**Affiliations:** ^1^School of Chemical Engineering and Food Science, Hubei University of Arts and Science, Xiangyang 441053, China; ^2^Key Laboratory of Low Dimensional Optoelectronic Materials and Devices of Hubei Province, Xiangyang 441053, China

## Abstract

Cu-Ni(OH)_2_/N-GR/Nafion/GCE has been prepared by electrodeposition and activation with NaOH. The proposed modified GCE was studied by electrochemical methods. It is found that dopamine shows favorable redox cyclic voltammetric response on the proposed modified GCE with peak separation of 25 mV and large current compared with on single-component modified GCE. The kinetic of electrode process has also been investigated with rate constant of 6.618 × 10^−3^ cm/s, which can be deduced to be a quasireversible or near-reversible process. The proposed method has been used for DA detection with linear range of 1.0 × 10^−7^ mol/L to 4.6 × 10^−5^ mol/L, and the detection limit is 3.3 × 10^−8^ mol/L. The proposed method has favorable stability and reproducibility and has also been used to determine DA in simulated samples and DA injections with favorable recoveries of 98.4% to 102.6%.

## 1. Introduction

Dopamine (DA), as one of the important neurotransmitters, plays a significant role in the function of the central nervous, renal, and hormonal systems. It has also been reported that low level of DA may result in some diseases such as Parkinson's disease and schizophrenia [1]. Therefore, the rapid and sensitive detection of dopamine is both necessary for diagnostic and pathological research [2]. For this reason, in the past several years, a variety of analytical techniques has been proposed for determination of dopamine in biological samples [3–5]. Among those methods, voltammetric techniques have attracted more and more interest for many researchers due to its quick and high response for the electrochemical oxidation of DA. The voltammetric methods mainly depend on the chemical modification of traditional electrode materials with self-assembled monolayer [6], metal and metal oxide nanoparticles [7, 8], carbon materials [9, 10], and conducting and/or electrochemically generated polymers [11, 12]. However, in biological samples electrochemical determination of dopamine is usually interfered by ascorbic acid and uric acid, and for this reason the selectivity of electrochemical sensors required for dopamine determination in biological samples is important. To solve this problem, several techniques have been used for anti-interfering of ascorbic acid, uric acid, and other interferents, such as Nafion film [10], charged self-assembled monolayers [13], and conducting polymers [14, 15].

Recently, a new two-dimensional (2D) carbon material, graphene (GR), attracts much attention in fabricating electrochemical biosensors [16, 17] because of its unique properties such as exceptional thermal and mechanical properties, large surface to volume ratio, and high electrical conductivity. DA has also been detected on graphene modified electrode. For example, Gao et al. [18] have reported that DA can be selectively determined on a graphene-chitosan nanocomposite modified electrode and the interference from AA can be completely eliminated by the modified layer with high sensitivity and selectivity.

Electrodes modified with gold, platinum, palladium, copper, nickel, and silver nanoparticles have revealed good performances in catalysis, facilitation of mass transport, and increase of effective surface area [19–22]. However, compared with noble metal nanoparticles and the other in situ preparation of electrodeposition nanoparticles, copper hydroxide and nickel hydroxide can be prepared cheaper and easier.

In this paper, nitrogen doped graphene was prepared and used to modified glassy carbon electrode and then copper and nickel oxide were electrodeposited onto the N-GR modified GCE. Using NaOH as electrolyte, the electrodeposited Cu/Ni oxide on the N-GR modified GCE can be transformed to be Cu/Ni hydroxide by cyclic voltammetric activation. The as-prepared electrode was used to investigate the electrochemical response of DA. It is found that DA can occur near-reversible electroreaction on this composite electrode, cyclic voltammograms of DA showed peak separation of 25 mV and symmetrical redox currents, and the kinetics of electrode reaction constant has been calculated to be 6.18 × 10^−3^ cm/s, which is similar to the rate constant of 10^−2^ cm/s. The proposed modified GCE has been used for DA with detection limit of 3.3 × 10^−8^ mol/L, favorable stability, reproducibility, and recoveries.

## 2. Experimental

### 2.1. Instruments and Chemicals

The nitrogen doped reduced graphene was prepared using a Teflon container with microwave. All the electrochemical experiments were carried out using CHI660A electrochemical workstation (USA) with three-electrode cell, modified GCE as working electrode, Ag-AgCl as reference, and Pt wire as counter electrode. The characterization of N-GR was confirmed at S-4800 SEM (Japan) and FTIR.

Dopamine, uric acid, ascorbic acid, sodium dodecyl benzene sulfonate (SDBS), and other chemicals were purchased from Shanghai Chemical Corporation (Shanghai, China), and all those are of analytical grade and used as received. Nafion (Aldrich). Phosphate buffer solution was prepared with NaH_2_PO_4_ and Na_2_HPO_4_; the pH value was adjusted by NaOH and H_3_PO_4_ to a suitable value.

### 2.2. Synthesis of N-GR

N-GR was synthesized according to the reference [23] with minor modifications. Briefly, the prepared graphene oxide was sonicated and dispersed for 1 h in pure water to keep its concentration of 2 mg/mL. Then the dispersed solution was centrifugally separated for 5 min at 1000 rpm to remove the unresolved GO residues. The as-prepared GO solution was adjusted to pH 10 with 30% aqueous ammonia, and 2 mL hydrazine hydrate was added and magnetically stirred for 10 min and then the mixed solution was transferred into a Teflon autoclave to react for another 3 h at 80°C with microwave. The synthesized N-GR was magnetically stirred, washed, and vacuum dried at 50°C. The prepared material was characterized by SEM and FTIR. Seen from the figures of N-GR, the sheets of N-GR fold together and frizzle on the edge of N-GR; this is the traditional feature of graphene. Comparing with the FTIR spectra of graphite oxide and N-GR, the spectra is much simple for N-GR, indicating that during the chemical reaction of hydrazine hydrate with graphite oxide, most of the groups containing oxygen have been removed and constructed graphene containing doped nitrogen, and the 1565 cm^−1^ absorption peak conferred the existence of C=C bond [24]; all those conferred the synthetic process of N-GR (figure not shown).

### 2.3. Preparation of Modified Electrodes

Preparation of N-GR Nafion modified GCE: 3 mg N-GR was added into 100 *μ*L 5% Nafion and 900 *μ*L 0.5% SDBS and magnetically stirred for 3 h to get a mixed suspension. Then 3 *μ*L of the suspension was dropped onto the cleaned GCE surface and dried.

Preparation of Cu-Ni(OH)_2_/N-RG/Nafion/GCE: the N-GR/Nafion GCE was dipped into 1.0 × 10^−3^ mol/L Cu^2+^ + 1.0 × 10^−3^ mol/L Ni^2+^ + 1.0 × 10^−4^ mol/L SDBS + 0.1 mol/L KCl (pH 5.0 ), three-electrode cell, and the modified GCE was handled with cyclic voltammetric sweeping from 1.2 V to −1.2 V for 20 scans at 100 mV/s. Then the as-prepared GCE was transferred into 0.3 mol/L NaOH for cyclic voltammetric scanning for 40 scans in the potential range of −0.2 V to 0.8 V at 100 mV/s. The modified GCE was labeled as Cu-Ni(OH)_2_/N-GR/Nafion/GCE. The other single-metal oxide modified GCE was prepared by the same procedure only by dipping Cu^2+^ or Ni^2+^-SDBS-KCl solution.

### 2.4. Electrochemical Investigation

The modified GCE was characterized by electrochemical impedance spectroscopy (EIS) in 0.1 mol/L KCl containing 1 mM Fe(CN)_6_^3−/4−^; the potential was set at 0.26 V in the frequency range from 0.01 Hz to 10^5^ Hz. In PBS with different pH values, the electrochemical behaviors of DA were investigated by cyclic voltammetry and differential pulse voltammetry (DPV). The DPV parameters are as follows: scan rate, 50 mV/s; amplitude, 0.05 V; and pulse width, 0.05 s. All the electrochemical experiments were carried out at room temperature (20 ± 2°C) and pure water was used throughout.

## 3. Results and Discussion

### 3.1. The Electrochemical Deposition of Cu-Ni Oxide Composite and Its Activation

**Figure 1:**
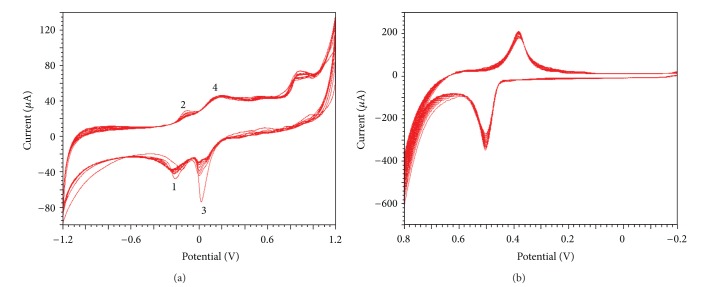
CVs of electrodeposition of Cu-Ni oxide (a). Electroactivation in NaOH (b) at 0.1 V/s.

Figure 1(a) is the cyclic voltammograms (CVs) of electrochemical deposition of Cu-Ni oxide composite onto N-GR/Nafion/GCE. As can be seen from the figure, with increasing of scanning number, the two pairs of peaks (labeled as 1 and 2, 3, and 4) increased gradually. Comparing with the CVs of mono-electrodeposition of Cu and Ni oxide, the peaks 1 and 2 can be attributed to the electrodeposition of Ni oxide, and peaks 3 and 4 can also be attributed to Cu oxide electrodeposition onto the N-GR/Nafion/GCE surface. The peaks 1 and 2 arose from the redox of Ni(III)/Ni(II) and the peaks 3 and 4 arose from the redox of Cu^+^/Cu^2+^ and Cu^2+^/Cu.

Figure 1(b) is the CVs of electrochemical activation of Cu-Ni oxide/N-GR/Nafion/GCE in 0.3 mol/L NaOH. As can be seen, a pair of redox peaks appeared at 0.48 V and 0.36 V, and this pair of peaks can be attributed to the redox of NiOOH/Ni(OH)_2_ [25]. However, the redox peaks shift a lot, and maybe the potential shifting is attributed to the generation of Cu(OH)_2_ at the composite surface, the potential shifts positively, and peak separation increased.

### 3.2. Electrochemical Characterization of Cu-Ni(OH)_2_/N-GR/Nafion/GCE

**Figure 2:**
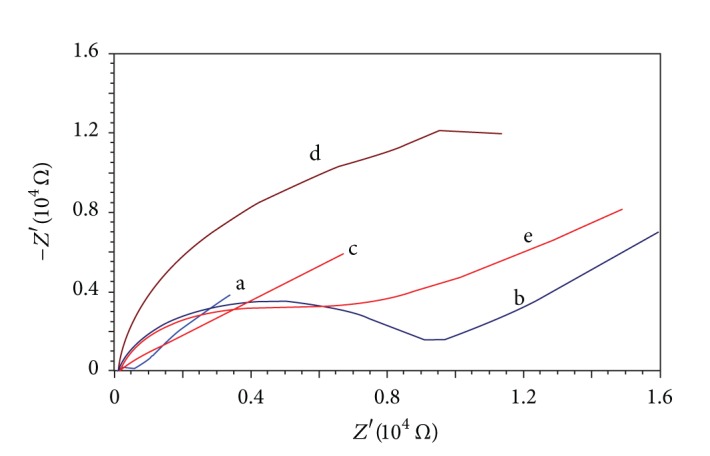
Nyquist plots of bare (a), N-GR/Nafion/GCE (b), Cu(OH)_2_/N-GR/Nafion/GCE (c), Ni(OH)_2_/N-GR/Nafion/GCE (d), and Cu-Ni(OH)_2_/N-GR/Nafion/GCE (e) in KCl containing 1 mmol/L Fe(CN)_6_^3−/4−^.

By using Fe(CN)_6_^3−/4−^ as redox probe, bare and modified GCE as working electrode, the EIS was utilized to character the surface of electrodes. As can be seen from Figure 2, the bare GCE can be observed with a little circle in high frequency and a line in low frequency at 45° (curve a). When GCE was modified with N-GR/Nafion, a large semicircle was observed in high frequency and a 45° line was observed in low frequency (curve b), as N-GR is a conducting carbon material, so the increase of resistance of charge transfer (*R*_ct_) may be attributed to the negatively charged Nafion film, which makes the Fe(CN)_6_^3−/4−^ difficult to reach GCE surface. When the N-GR/Nafion/GCE was further electrodeposited Cu(OH)_2_, the Nyquist plot can be seen with a line in all frequency (curve c); this phenomenon may be explained that electrodeposited Cu(OH)_2_ on N-GR/Nafion film counteracts effectively the negatively charged Nafion film and built some charge transfer passage to facilitate the approaching of Fe(CN)_6_^3−/4−^. While N-GR/Nafion/GCE was electrodeposited with Ni(OH)_2_, a huge semicircle was observed (curve d), indicating that Ni(OH)_2_ film deposited onto N-GR/Nafion film further hinder the approach of probe ions to the electrode surface. Comparing with the Nyquist plots of electrodeposition of Cu(OH)_2_, Ni(OH)_2_ and Cu-Ni(OH)_2_ onto N-GR/Nafion/GCE, it can be seen that the Nyquist plot of Ni(OH)_2_/N-GR/Nafion/GCE (curve e) has a *R*_ct_ (larger than N-GR/Nafion/GCE and lower than Ni(OH)_2_/N-GR/Nafion/GCE), indicating that the co-deposited Cu(OH)_2_ still built some charge transfer passage in the composite Cu-Ni(OH)_2_ film, and the codeposition of Cu-Ni(OH)_2_ facilitates the electron transfer and redox reaction of Fe(CN)_6_^3−/4−^ on the electrode surface. All the EIS results are in accordance with those of cyclic voltammetry.

### 3.3. Electrochemical Behavior of DA on Different Electrodes

**Figure 3:**
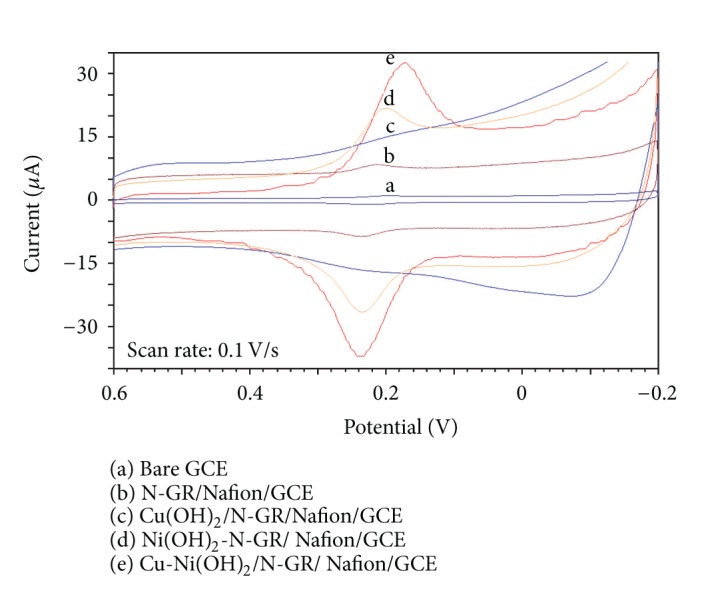
CVs of 0.03 mmol/L DA on different electrodes in pH 6.5 PBS.

The electrochemical behaviors of DA at different electrodes were investigated in PBS (pH = 6.5) in the potential of −0.2 V to 0.6 V (Figure 3). As can be seen, no redox peaks can be observed at Cu(OH)_2_/N-GR/Nafion/GCE (curve c), indicating that the electrooxidation of DA can be hindered on this film, maybe the retardation of low electroactive copper hydroxide towards the electrooxidation of DA, while on the bare GCE, N-GR/Nafion/GCE, Ni(OH)_2_/N-GR/Nafion/GCE, and Cu-Ni(OH)_2_/N-GR/Nafion/GCE, a pair of redox peaks can be observed with different peak currents and peak separation. Comparing with the four cyclic voltammograms, very little currents (0.53 *μ*A) and unproducible CVs can be seen at bare GCE, indicating that DA undergoes surface passivation process at bare GCE. When N-GR was modified onto GCE, the cyclic voltammetric current increased to 1.963 *μ*A, indicating the electrocatalytic behavior of N-GR towards DA, but the electrocatalytic current is only four times than that of bare GCE. Further modification of Ni(OH)_2_ onto N-GR/Nafion film, large peak current (11.02 *μ*A), and low peak separation (Δ*E*_p_ = 56 mV) can be seen, which indicated that Ni(OH)_2_ and N-GR can effectively electrocatalyze the electrooxidation of DA; it is obvious that electrocatalytic activity towards DA arises from electrodeposited Ni(OH)_2_. However, the codeposition of Cu-Ni(OH)_2_ onto N-GR/Nafion/GCE, a pair of reproducible and symmetrical redox peaks can be seen with large peak current (23.74 *μ*A); *E*_pa_ = 0.238 V and *E*_pc_ = 0.213 V and low peak separation (Δ*E*_p_ = 25 mV). The possible explanation of Ni(OH)_2_ film maybe the electrocatalysis of Ni(III)/Ni(II) towards the electrooxidation of DA, and coelectrodeposition of Cu-Ni(OH)_2_, and the copper hydroxide can build conducting passage on the composite film, facilitating the electron transfer between DA, which is similar to the process in the previous cyclic voltammetric and EIS experiments. The strong electrocatalysis of codeposition of Cu(OH)_2_-Ni(OH)_2_ towards DA may be attributed to the facilitating of electron transfer and electrocatalysis of NiOOH/Ni(II) towards electrooxidation of DA.

### 3.4. Influence of Supporting Electrolyte and pH Value

Supporting electrolyte can affect the electrochemical properties of electrode and the electrochemical reaction of analytes. In this experiment, different electrolytes (PBS, Britton-Robinson, HCl, HAc, NaAc-HAc, KCl) with different pH values (pH = 6.0 to 8.5) have been used to investigate the electrochemical behavior of DA at Cu-Ni(OH)_2_/N-GR/Nafion/GCE. It is found that the electrochemical behavior of DA showed favorable redox properties in PBS, so in the latter experiments, 0.1 mol/L PBS was chosen as the supporting electrolyte.

**Figure 4:**
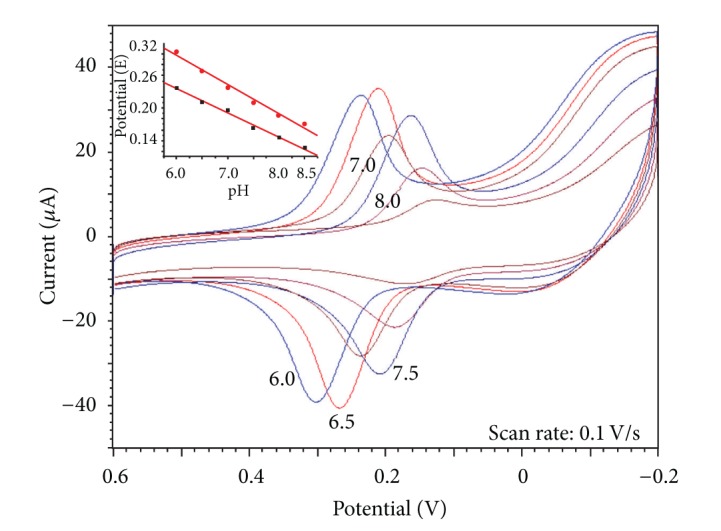
CVs of 0.3 mmol/L DA on Cu-Ni(OH)_2_/N-GR/Nafion/GCE in PBS with different pH.

The effects of pH values of electrolyte on the electrochemical behaviors of DA have also been investigated.  Figure 4 is the CVs of 0.03 mmol/L DA at different pH values (from 6.0 to 8.0), and the inset graph is the peak potential versus pH. It is found that the peak currents are the largest in pH 6.5 PBS, and the redox peak potential is linear to pH, and the regression equations are *E*_pa_(V) = 0.6146 − 0.05314  pH, *R* = 0.9923; *E*_pc_(V) = 0.5042 − 0.04474  pH, *R* = 0.9961. The slopes of two linear equations are close to the slope about “two protons and two electrons mechanism” (55.8 mV/pH), indicating that DA in this electrode underwent an electrochemical process of two protons and two electrons [26].

### 3.5. Effect of Scan Rate on the Electrochemical Behavior of DA

**Figure 5:**
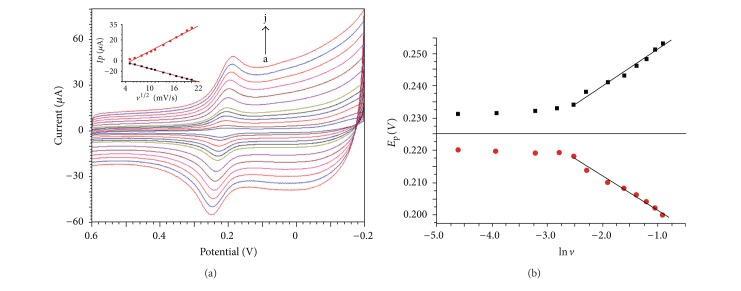
(a) CVs of 0.03 mmol/L DA at Cu-Ni(OH)_2_/N-GR/Nafion/GCE at different scan rates, inset figure is the *I*_*p*_ versus *ν* ^1/2^. (b) Peak potential versus ln⁡*ν* (b).

Figure 5(a) is the CVs of 0.03 mmol/L DA at different scan rate. As can be seen, the peak current is linear to the square root of scan rate with regression equations of *I*_pa_ = 0.7839 − 0.9538*ν* ^1/2^, *R* = 0.9994; *I*_pc_ = −6.268 + 1.826*ν* ^1/2^, *R* = 0.9949. The results indicate that the electrochemical behavior of DA is diffusion-controlled.

In a further investigation of the effect of scan rate on peak potential, it is found that the redox peak potential is linear to the natural logarithm of scan rate with the regression equation of *E*_pa_ = 0.2619 + 0.011ln⁡*ν*, *r* = 0.9896; *E*_pc_ = 0.1915 − 0.010ln⁡*ν*, *r* = 0.9893. Based on the quasireversible current theory [27], Wang et al. have calculated the diffusion coefficients of DA by steady state voltammetry (*D* = 4.9 × 10^−6^ cm^2^/s) [28]. According to [29],

(1) Epa=Eθ+m[0.78+ln⁡Dks−0.51ln⁡ m]+m2ln⁡νm=RT(1−α)nαF,Epc=Eθ−m′[0.78+ln⁡Dks−0.51ln⁡ m′]−m′2ln⁡νm′=RTαnαF.

Based on the slope of the regression equation, (1 − *α*)*n*_*α*_ = 1.168, *n*_*α*_ *α* = 1.284 can be calculated. When *a* = 0.4 to 0.6, then *n* can be calculated to be 2; this indicates that the two-electron transfer is processed in one step. And rate constant of electrode reaction of DA can also be calculated to be *k*_*s*_ = 6.627 × 10^−3^ cm/s and *k*_*s*_ = 6.609 × 10^−3^ cm/s; the average value is 6.618 × 10^−3^ cm/s. According to the kinetics of electrode process, when the rate constant is larger than 10^−2^ cm/s, the electron transfer process is very fast, and the electrode reaction is reversible, and when 10^−4^ < *k*_*s*_ < 10^−2^ cm/s, the electrode reaction is a quasireversible process. So the electrochemical reaction of DA on the proposed electrode is a quasireversible process or near reversible process, which is the synergistic electrocatalysis of N-GR and Cu-Ni(OH)_2_ composite film to lead the quasireversible or near-reversible electrooxidation of DA.

### 3.6. Determination of DA

**Figure 6:**
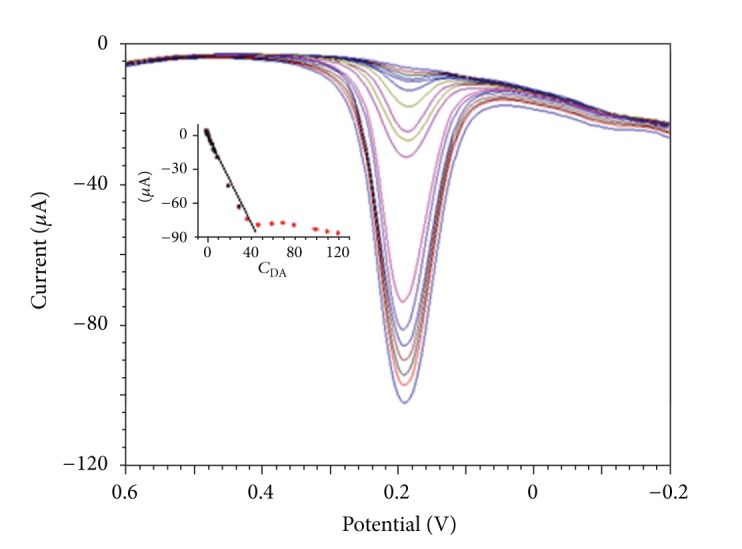
DPVs of DA with different concentration on Cu-Ni(OH)_2_/N-GR/Nafion/GCE, inset picture is the *I*_pa_ versus *C*_DA_.

The proposed modified GCE has the favorable electrochemical response towards DA, so it has been used for determination of DA. To improve the sensitivity of the proposed method in detection of DA, differential pulse voltammetry (DPV) has been used. It is found that the electrochemical oxidation peak DPV current is linearly proportional to its concentration in the range from 1.0 × 10^−7^ mol/L to 4.6 × 10^−5^ mol/L with the regression equation of *I*_*p*_ (*μ*A) = −0.5812 − 2.148*c* (*μ*mol/L), *R* = 0.9981, respectively, (Figure 6). The detection limit can be calculated to be 3.3 × 10^−8^ mol/L (*S*/*N* = 3).

### 3.7. Stability, Reproducibility, and Accuracy of Cu-Ni(OH)_2_/N-GR/Nafion/GCE

To further investigate of the stability, reproducibility, and accuracy of the proposed modified GCE towards DA, cyclic voltammetric scanning of the proposed modified GCE towards the electrochemical behavior of 3 × 10^−5^ mol/L DA has been accomplished. It is found that the redox currents and peak potentials keep almost unchanged after 20 scans; the relative standard deviation (RSD) of the currents is 2.1%, which indicates that the proposed modified GCE has favorable reproducibility.

The stability of the modified GCE was evaluated by the electrochemical response of 3 × 10^−5^ mol/L DA determined by one modified GCE in one month. It is found that the electrochemical response of DA keeps almost unchanged in one week and decreases about 5.6% in one month. This indicates that the proposed modified GCE has excellent stability. Successive modification of 5 GCEs with similar technique and investigate cyclic voltammetric response towards DA, the RSD is 4.5%, indicating the stability of the proposed modified GCE is favorable and suit for bulk preparation.

The accuracy of the proposed modified GCE was evaluated by determination of DA content by standard addition method. The proposed GCE was applied to determine the content of DA with known concentrations in water and the DA drug injections. The developed electrode exhibited exact recovery results between 98.4% and 102.6% by standard addition method. To illustrate the feasibility of proposed modified GCE for routine analysis, the electrode was applied to determine DA in dopamine hydrochloride injection solution (10.0 mg/mL DA, 2.0 mL per injection). Each sample was detected several times repeatedly. Results showed that the average value of the injection was about 9.89 mg/mL with RSD of 2.7%, which was in accordance with the standard content. The satisfactory results obtained with Cu-Ni(OH)_2_/N-GR/Nafion/GCE indicated that the proposed method can be applied to real sample assay.

### 3.8. Interference of Other Substances

Under the optimum conditions, the effects of some coexisting interferents upon the determination of 0.03 mmol/L DA have also been investigated (Table 1). The results show that 100 times of Cl^−^, NO_3_^−^, K^+^, Mg^2+^, Zn^2+^, Na^+^, 100 times of AA and H_2_O_2_, and 1000 times of glucose have almost no influence on the electrochemical response of 0.03 mmol/L DA. But 100 times of uric acid will bring out big interference to the detection of 0.03 mmol/L DA. This result shows that the proposed method has favorable anti-interference ability.

## 4. Conclusions

Cu-Ni(OH)_2_/N-GR/Nafion/GCE has been prepared by electrodeposition and activation with NaOH. The proposed modified GCE was studied by electrochemical methods. It is found that dopamine shows favorable redox cyclic voltammetric response on the proposed modified GCE with peak separation of 25 mV and large current compared with on single-component modified GCE. The kinetic of electrode process has also been investigated with rate constant of 6.618 × 10^−3^ cm/s, which can be deduced to be a quasireversible or near-reversible process. The proposed method has been used for DA detection with linear range of 1.0 × 10^−7^ mol/L to 4.6 × 10^−5^ mol/L; the detection limit is 3.3 × 10^−8^ mol/L. The proposed method has favorable stability and reproducibility and has also been used to determine DA in simulated samples and DA injections with favorable recoveries of 98.4% to 102.6%.

## Figures and Tables

**Table 1:** Influence of coexisting substances on the determination of 0.03 mmol L^−1^ DA (*n* = 3).

Coexisting substance	Concentration ratio of dopamine : interferent	Current ration^a^ (%)^a^
Zn^2+^	1 : 100	0.993
K^+^	1 : 100	1.01
Cl^−^	1 : 100	0.985
NO_3_^−^	1 : 100	0.997
Na^+^	1 : 100	1.01
Mg^2+^	1 : 100	0.983
Glucose	1 : 1000	1.04
Ascorbic acid	1 : 100	1.04
Uric acid	1 : 100	0.465
H_2_O_2_	1 : 100	0.977

^a^The current ratio is defined as the ratio of currents obtained from 0.03 mmol L^−1^ DA with and without interferent.

## References

[1] Wightman RM, May LJ, Michael AC (1988). Detection of dopamine dynamics in the brain. *Analytical Chemistry*.

[2] Zen J-M, Chen P-J (1997). A selective voltammetric method for uric acid and dopamine detection using clay-modified electrodes. *Analytical Chemistry*.

[3] Naiaraia P, Murthy KCS, Rangappa KS, Gowda NM (1998). Spectrophotometric methods for the determination of certain catecholamine derivatives in pharmaceutical preparations. *Talanta*.

[4] Deftereos NT, Calokerinos AC, Efstathiou CE (1993). Flow injection chemiluminometric determination of epinephrine, norepinephrine, dopamine and L-dopa. *Analyst*.

[5] Oztekin Y, Ramanaviciene A, Yazicigil Z, Solak AO, Ramanavicius A (2011). Direct electron transfer from glucose oxidase immobilized on polyphenanthroline-modified glassy carbon electrode. *Biosensors and Bioelectronics*.

[6] Liu C, Lu G, Jiang L, Jiang L, Zhou X (2006). Study on the electrochemical behavior of dopamine and uric acid at a 2-amino-5-mercapto-[1,3,4] triazole self-assembled monolayers electrode. *Electroanalysis*.

[7] Liu C, Hu J, Hu J, Tanga H (2006). Electrocatalytic oxidation of dopamine at a nanocuprous oxide-methylene blue composite glassy carbon electrode. *Electroanalysis*.

[8] Oztekin Y, Tok M, Bilici E (2012). Copper nanoparticle modified carbon electrode for determination of dopamine. *Electrochimica Acta*.

[9] Palanisamy S, Ku S, Chen S-M (2013). Dopamine sensor based on a glassy carbon electrode modified with a reduced graphene oxide and palladium nanoparticles composite. *Microchimica Acta*.

[10] Yang S, Li G, Yin Y, Yang R, Li J, Qu L (2013). Nano-sized copper oxide/multi-wall carbon nanotube/Nafion modified electrode for sensitive detection of dopamine. *Journal of Electroanalytical Chemistry*.

[11] Chen J, Zhang J, Lin X, Wan H, Zhang S (2007). Electrocatalytic oxidation and determination of dopamine in the presence of ascorbic acid and uric acid at a poly (4-(2-pyridylazo)-resorcinol) modified glassy carbon electrode. *Electroanalysis*.

[12] Kausaite-Minkstimiene A, Mazeiko V, Ramanaviciene A, Ramanavicius A (2011). Evaluation of amperometric glucose biosensors based on glucose oxidase encapsulated within enzymatically synthesized polyaniline and polypyrrole. *Sensors and Actuators B*.

[13] Rai CR, Ohsaka T (2001). Electrocatalysis of ascorhate and dopamine at a gold electrode modified with a positively charged self-assembled monolayer. *Journal of Electroanalytical Chemistry*.

[14] Qian T, Yu C, Wu S, Shen J (2013). In situ polymerization of highly dispersed polypyrrole on reduced graphite oxide for dopamine detection. *Biosensors and Bioelectronics*.

[15] Shaidarova LG, Gedmina AV, Artamonova ML, Chelnokova IA, Budnikov HC (2013). Voltammetry determination of dopamine by the electrocatalytic response of an electrode modified by a polyaniline film with an inclusion of copper(II) tetrasulfophthalocyanine. *Journal of Analytical Chemistry*.

[16] Xu H, Wang D, He S (2013). Graphene-based nanoprobes and a prototype optical biosensing platform. *Biosensors and Bioelectronics*.

[17] Chen X, Jia X, Han J, Ma J, Ma Z (2013). Electrochemical immunosensor for simultaneous detection of multiplex cancer biomarkers based on graphene nanocomposites. *Biosensors and Bioelectronics*.

[18] Gao F, Cai X, Wang X (2013). Highly sensitive and selective detection of dopamine in the presence of ascorbic acid at graphene oxide modified electrode. *Sensors and Actuators B*.

[19] Welch CM, Compton RG (2006). The use of nanoparticles in electroanalysis: a review. *Analytical and Bioanalytical Chemistry*.

[20] La D-D, Kim CK, Jun TS (2011). Pt nanoparticle-supported multiwall carbon nanotube electrodes for amperometric hydrogen detection. *Sensors and Actuators B*.

[21] Atta NF, Galal A, Ahmed RA (2011). Poly(3,4-ethylene-dioxythiophene) electrode for the selective determination of dopamine in presence of sodium dodecyl sulfate. *Bioelectrochemistry*.

[22] Palanisamy S, Ku S, Chen S-M (2013). Dopamine sensor based on a glassy carbon electrode modified with a reduced graphene oxide and palladium nanoparticles composite. *Microchimica Acta*.

[23] Long D, Li W, Ling L, Miyawaki J, Mochida I, Yoon S-H (2010). Preparation of nitrogen-doped graphene sheets by a combined chemical and hydrothermal reduction of graphene oxide. *Langmuir*.

[24] Seredych M, Hulicova-Jurcakova D, Lu GQ, Bandosz TJ (2008). Surface functional groups of carbons and the effects of their chemical character, density and accessibility to ions on electrochemical performance. *Carbon*.

[25] Vais RD, Sattarahmady N, Heli H (2013). A Nanocomposite of nickel hexacyanoferrate dots-graphene nanosheets-applied to the electrocatalytic oxidation and determination of N-acetyl-L-cysteine. *Sensor Letters*.

[26] Huang Y, Cheng CM, Tian XQ (2013). Low-potential amperometric detection of dopamine based on MnO_2_ nanowires/chitosan modified gold electrode. *Electrochim Acta*.

[27] Wu ZB, Zhang ZX (1992). Linear sweep voltammetric investigation (III)-Quasi-reversible current theory controlled by diffusion and electrode reaction on microelectrodes. *Chemical Journal of Chinese Universities*.

[28] Wang Q, Jiang N, Li N (2001). Electrocatalytic response of dopamine at a thiolactic acid self-assembled gold electrode. *Microchemical Journal*.

[29] Aoki K, Tokuda K, Matsuda H (1987). Theory of stationary current-potential curves at microdisk electrodes for quasi-reversible and totally irreversible electrode reactions. *Journal of Electroanalytical Chemistry*.

